# Using App Store Reviews to Explore User Experiences and Perceptions of Mental Health Mobile Apps: Qualitative Study

**DOI:** 10.2196/97517

**Published:** 2026-10-08

**Authors:** Rachana Talekar, Israel Arevalo, Blanca Macareno, Whitney Garney

**Affiliations:** 1 Telehealth Institute, Texas A&M Health Science Center, 1700 Research Parkway, College Station, TX, 77840, United States, 1 (979) 436 0700; 2 School of Public Health, Texas A&M Health Science Center, 212 Adriance Lab Rd, College Station, TX, 77843, United States, 1 (979) 436-9443; 3 Center for Community Health & Aging, School of Public Health, Texas A&M Health Science Center, College Station, TX, United States

**Keywords:** mental health, user experience, mobile health, apps, anxiety, depression, qualitative

## Abstract

**Background:**

With the rise of digital health, mental health apps (MHapps) have become an increasingly popular tool in the self-management of depression, anxiety, and stress. Star ratings are often used as indicators of app quality to guide selection. However, ratings do not fully capture if the MHapp’s features, usability, or design elements exhibit the heterogeneous real-life user experiences. Instead, examining the patterns of engagement and app usage can highlight whether high ratings reflect diverse user needs.

**Objective:**

This study aimed to examine user experience of top-rated depression and anxiety apps to assess if user perceptions, as reported in app reviews, align with star ratings.

**Methods:**

To identify relevant mobile apps, we used a systematic search strategy that mimicked a typical user’s journey and applied search terms related to depression, anxiety, and stress management. Apps were screened using a predefined inclusion criteria that guided the identification of the 5 most highly rated MHapps, yielding approximately 750 user reviews across all apps. Reviews were analyzed using hybrid inductive and deductive coding techniques, with codes generated iteratively to capture the spectrum of user perceptions and experiences. Codes were then clustered, leading to theme generation that provided a deeper understanding of user experiences.

**Results:**

User experiences demonstrated a complex interplay between perceived benefits and frustration. Users valued ease of navigation, convenience, accessibility, and low-effort features that allowed easier entry to self-management of mental health. Users expressed their affinity toward specific app features and desire for the addition of new features that could further support their mental wellness. However, limited or restrictive features due to paywall, in-app advertisement placement, and functionality issues resulting from technical issues or bugs garnered substantial criticism.

**Conclusions:**

This study highlights the complexity of user perceptions influencing experience with self-management MHapps. Despite high star ratings, app usability elicited paradoxical responses and showed multiple overlapping themes, showcasing features that may support some users but hinder others. The results emphasize that star ratings alone are insufficient to showcase if a MHapp meets user needs. Continued improvement in app design such as interface usability, feature development, and pricing transparency is needed to meet the diverse needs of users for better engagement and satisfaction.

## Introduction

Digital technologies are driving a revolutionary shift in health care by providing more accessible and efficient forms of services and resources [1]. The mobile health (mHealth) app market provides users with quick and easy methods of engagement to modify health behaviors [2]. These apps allow instantaneous access, which speeds up the transfer of health information [3]. With smartphone ownership increasing to almost 91% among Americans, these devices have become crucial for everyday living, including managing individual health [4]. mHealth is opening new avenues for health care providers, researchers, and the public by improving health monitoring, increasing awareness of health risks, and facilitating access to prevention and support services [5,6].

Due to high demand, the mental health sector has embraced mobile technology, leading to a surge in mental health innovations [7]. This convergence of mental health needs and digital solutions not only expands access to care but also redefines how mental health services are delivered, making support more immediate, personalized, and user-driven. Today, over 20,000 mental health apps (MHapps) have emerged to provide convenient solutions that bridge care gaps created by physical distancing and an overburdened mental health care workforce [8-11]. For example, a study in a psychiatry outpatient setting showed that 69% of respondents, and 80% of those younger than 45 years of age, wanted to use a mobile app to track their mental health [12]. Such studies signal user readiness and appetite for tools to support self-management of health.

Support via MHapps can range widely—from basic text messaging and crisis helplines to interventions using wearable devices [13-15]. Several apps include evidence-based treatment approaches such as cognitive behavioral therapy (CBT), mindfulness training, mood monitoring, and cognitive skills training to address depressive symptoms [16]. The ease of use, low effort expectancy, and high hedonic motivation make mobile apps a viable choice for delivering mental health support [7]. Yet, the promise and consumption of MHapps have outpaced the available evidence on such tools. Most effectiveness data are tied to development-stage refinements focusing on functionality and appeal to consumers and limit focus on evaluating user experience with clinical utility [17,18]. Additionally, MHapps evaluated in randomized controlled trials are often not publicly available, while those available in the public domain have limited evidence [19,20]. As interest in MHapps has grown, the literature has reflected the trajectory and expanded from early studies focusing on acceptability to more recent studies examining real-world interaction with MHapps [20-23].

However, findings on MHapps’ impact have been mixed, with inconsistencies on the impact of such apps, ranging from some recommended tools to “cannot recommend” as a stand-alone psychological intervention for the treatment of depression and anxiety [24-26]. To ensure safe and effective digital mental health care, it is essential to scientifically assess the advantages and limitations of unguided and public “in the wild” available MHapps [18]. Furthermore, many MHapps’ adherence to best practices in both clinical and data privacy is muddled, thus increasing concerns on misinformation, disinformation, or confidentiality breaches [27,28]. Currently, fewer than 5% of more than 20,000 available MHapps are supported by empirical research, and only a handful have evidence to support their effectiveness [24]. While the digital field is evolving, the utility and quality of apps continue to be insufficiently assessed [8,29].

Adding to these concerns, MHapps differ in their intended user journeys, making retention a heterogeneous concept. Some apps are designed for daily use, while others are more emergent with sporadic use. Industry views app success in limited terms such as user engagement, sustainability, and app retention [30-32]. Such limited framing can obscure the depth in user-level experience and how individuals interact with and value the components offered by different MHapps [33,34]. This compounds a critical need to understand user-level characteristics of retention and engagement and their association with app-level characteristics.

Centering on user experience as an assessment of quality and driver of real-world effectiveness, the purpose of this study is to expand the digital mental health literature to include in-depth user experiences, perceived value, and user-centric implementation considerations among open-access MHapps in the iOS system, Apple’s mobile operating system. The study sought to answer the following research questions:

- (1a) How does a virtual user interact with apps designed for depressive or anxiety-like symptoms?
- (1b) What factors encourage or discourage users’ motivation to engage with the apps?
- (2) How does a virtual user assess the effectiveness of an app that is advertised to manage depressive or anxiety-like symptoms?

## Methods

### Research Design

This study uses an interpretative qualitative design to examine user reviews posted on the App Store and assess user engagement for highly rated MHapps. Interpretative research assumes social reality is not objective but shaped by human experience [35]. Surrounded by this social reality, interpretative research is a “sense-making” process over hypothesis testing [36]. Underscoring that increased engagement does not always result in better outcomes, there is a growing emphasis on assessing objective and subjective measures [37]. User experience plays a critical role in key parameters such as user acceptance and usability feedback, expanding research from quantifying attributes to user-centric processes that explore interaction among technology-human depth [38,39]. Additionally, to construct an explanation that considers the lived experiences, this study uses grounded theory to systematically analyze user-generated app reviews, using sets of procedures based on coding processes and theoretical sampling to construct a model or theory [40,41]. The research team continued coding and theme development iteratively until no new interpretive insight was generated. Analysis focuses on reflexive engagement with the data, ensuring rigorous interpretation of user reviews [42]. Hence, interpretative research design was an effective choice to capture social phenomena focusing on language, signs of engagement, and meaningful perspectives of users that otherwise may not be captured using positivist research techniques. This approach was well-suited for the study to explore MHapps research where user behavior intersects with perceptions of utility and context dependence.

The research team’s background included public health, community health, clinical psychology, computer-human interaction, and systems design expertise that informed the data collection and analysis process. The interdisciplinary interpretation allowed a broader understanding of the user-generated content that acknowledged the variance in emerging themes. Debriefing sessions conducted at regular intervals during data analysis supported reflexivity as external interpreters. Researcher positionality and transparency were maintained throughout by critique analysis of results, in which researchers not directly involved in coding reviewed the emerging themes.

### App Selection

The iOS system’s Apple App Store was selected as the sampling platform because its interface prioritizes highly rated and “Editor’s Choice” apps in search results, which align with the common user decision-making process. Additionally, the iOS system has a higher user market in the United States; hence, consumers may gravitate toward this due to the perceived credibility of star ratings [43]. The rationale for using higher-rated apps is to take into consideration the human thought process when deciding which app to download. This may include human behavior, intention, and preference given to the higher-rated apps or demotivation to scroll through too many options and make conclusive decisions from the first few mobile apps that may be available with higher ratings [44].

The app search was conducted using commonly identifiable, mental health–related keywords, such as “depression,” “anxiety,” “panic attacks,” and “mental health.” These terms are used interchangeably in app descriptions and reviews. A search limitation was added to limit apps to depression and anxiety assistive tools only, to ensure that the study accurately delivered information based on user engagement and designer intention.

Criterion sampling along with maximum variation was simultaneously used for the sampling strategy. Keyword searches were restricted to major terms, that is, depression, anxiety, panic attacks, and mental health. For each keyword, the top 10 apps that surfaced on the App Store with all the following criteria fulfilled were included in the sampling frame: (1) more than 10,000 ratings; (2) average rating of at least 4.5; and (3) software updates have been conducted in at least the last 6 months. Subsequently, the sample selection was limited to the top 5 apps fitting under at least one of the categories (maximum variations sampling): (1) coping tools or mechanisms or direct service delivery, (2) journaling, (3) interactive and based on positive reinforcement, and (4) individual or group motivation. It is important to note that the selection criteria apply only to identify eligible apps and not to the reviews analyzed within them. Hence, even a highly rated app may have a variety of user reviews ranging from strongly positive to possibly critical. Apps with extensive commercial advertisements such as Calm (Calm.com, Inc) and Headspace (Headspace Inc) were excluded. This study relies on App Store reviews as a proxy to understand user experience. Thus, including commercially advertised apps (eg, Calm and Headspace), whose reviews may be shaped by its marketing infrastructure, may introduce data biases and undermine the organic engagement with apps. Additionally, apps like Calm and Headspace are broader wellness apps. While they may be widely downloaded as meditation or stress reduction apps, they do not exclusively target depression and anxiety. Hence, including them in the study of app usage risks mixing apps focusing on stress-relief management in addition to targeted depression or anxiety coping mechanisms.

### Data Collection

Data scraping has been used for many years as an open-source database and for various purposes—the start of which was a Twitter (Twitter, Inc; subsequently rebranded as X) repository database [45]. Following app selection, user reviews were collected from the publicly accessible Apple App Store. The data scraping was enabled using Python (3.12, Python Software Foundation) and the Python library *app-store-scraper* developed by Eric Lim [46]. The *app-store-scraper* library allows for the retrieval of user reviews from the Apple App Store using an app name, country code, and optional app ID. It can fetch reviews in batches; filter by date; and return structured review data such as rating, review text, title, date, username, and edit status. Researchers built a custom data pipeline that ingests raw data scraped from the App Store (*app-store-scraper* output), wrangled the data for further analysis, and compiled it into a Microsoft Excel file. Given the inclusion criterion, reviews posted between January and August 2024 were included in the analysis, resulting in over 750 reviews that were coded and analyzed.

### Ethical Considerations

For this specific research subject, the data collected only included publicly posted user reviews, and no content from closed or interactive forums with an expectation of confidentiality or privacy was investigated. Measures were taken to ensure no identifiable or unpublished data that compromised the individual identity of the members participating in the activities on the App Store were scraped. For example, the information scraped was reviewed and matched with information available online. The legality of web scraping remains a gray area, and regulations have yet to develop to address big data directly [47]. For this study, data scraping methods were reviewed by a computer science expert to ensure that the code did not extract any identifiable information without the knowledge of researchers. As of now, web-scraping is guided by a set of related fundamental laws such as copyright infringement and the Computer Fraud and Abuse Act (CFAA) [48]. The researchers applied measures to ensure identifiable data were not extracted and the data collection did not require any interaction with individuals. Hence, no informed consent was needed for this phase of the study. Taking into consideration the criteria proposed by Eysenbach and Till [49], the study did not require informed consent [49]. Additionally, this study was conducted under the supervision of Texas A&M University faculty, and institutional review board (IRB) approval was not required per the university’s Standard Operating Procedures Human Research Protection (SOP HRP-093), as the study did not involve human research subjects and used only publicly available open-source data [50].

### Analysis

Moving beyond the lens of star-rating systems and focusing on experiences and usage patterns described in user reviews, the analysis captures nuanced insights into the effect and influence of MHapps across social interactions that may unfold among its users. Interpretative research involves rigorous and iterative stages to explore multifaceted social interactions [51]. Hence, its effectiveness is at its best when studies are within the sociohistoric context and subjectively interpret various epistemologies or participants [36]. Therefore, for this study, interpretative research design helped capture the social phenomenon focusing on language, signs of engagement, and meaningful perspective of virtual users’ engagement with MHapps that otherwise may not be captured using statistical techniques used by positivist research. A hybrid approach was used to conduct a comprehensive thematic analysis of the 750 user reviews for 5 apps aimed at alleviating depression, anxiety, and stress. This method combines inductive coding, through which themes developed through reflexive engagement with the data, with deductive coding, thus using predefined codes grounded in established theories. By fusing these 2 approaches, the research sought to unearth critical factors influencing user experience and satisfaction. This integrated methodology not only anchored the analysis of existing knowledge but also paved the way for fresh insights, ultimately shaping the development of more effective and user-centric MHapps.

User review analysis was done through a manual line-by-line coding process to preserve sensitivity to tone and emotional meaning, which were critical in understanding user sentiment and coding appropriately. Reviews were organized by year and month in Microsoft Excel to manage the volume and allow systematic temporal comparison, progressing from the most recent to older reviews. Two qualitative researchers (RT and BM) independently refined and clustered codes. After coding processes, the theme categories were developed by them, and any divergence in coding scheme was resolved through direct 1:1 discussion until consensus was reached. An initial codebook was developed through a 2-stage process. One researcher (RT) conducted preliminary coding of one of the app’s most recent month’s user reviews to develop preliminary patterns and establish a coding framework. This preliminary work was reviewed by the second researcher (BM) to ensure the derived codes incorporated inductive and deductive codes and were based on the framework of users’ engagement and experience. Both researchers (RT and BM) engaged in structured training on using the codebook and coded shared subsets. Discrepancies were discussed via regular meetings to ensure consensus in codes. This allowed the team to synthesize coherent themes and subthemes. Theme generation was assessed iteratively as coding clusters progressed temporally, and analysis continued for all user reviews within the sample until all codes sufficiently converged into existing themes and no new distinctions surfaced for researchers across all clustered codes. The themes, subthemes, and example codes’ structure are included as Multimedia Appendix 1. This supported the research intention of capturing the spectrum of user perceptions and experiences.

Trustworthiness evaluation in qualitative research can be conducted through credibility, transferability, dependability, and confirmability factors [52]. The study aligned these mechanisms through iterative engagement of themes across 5 apps, ensuring findings reflected user perspective and effectively answered research questions. Credibility was ensured by data familiarization and triangulation of themes. The research team maintained individual memos and engaged in critical discussions at key stages in data analysis to acknowledge any differing interpretations. Transferability was addressed in this study through the description of the app selection process, selection criteria, data scraping process, and extraction fields. The documented codes and cross-verification among researcher interpretations establish dependability. Lastly, the use of verbatim excerpts of user-generated reviews supports confirmability, ensuring that findings reflect users’ perception and not researcher projection. Thus, using reflexivity and trustworthiness techniques during stages of coding and theme generation ensured the researchers to be conscious and transparent about the influence of their academic and professional backgrounds throughout the coding and theme generation phase.

## Results

### Overview

Following the inclusion criteria (Figure 1), 5 MHapps were retained for analysis. The following apps were included in the final sample: Iam (Monkey Taps), Feelsy (PLAY4FUN APPS), Daylio (Relaxio s.r.o.), Daily Bean (BlueSignum Corp.), and Motivation (Monkey Taps). Each app presented a range of interactions with users, including but not limited to affirmations, relaxation prompts, mood tracking, progress tracking, symptom reporting, journaling, and motivational prompts. The results presenting the distribution of star ratings and average ratings for each app are shown in Figures 2 and 3. Notably, given that the highly rated apps consisted of a spectrum of user reviews, the line-by-line coding of these reviews revealed both factors that encouraged as well as discouraged engagement among users.

**Figure 1.**
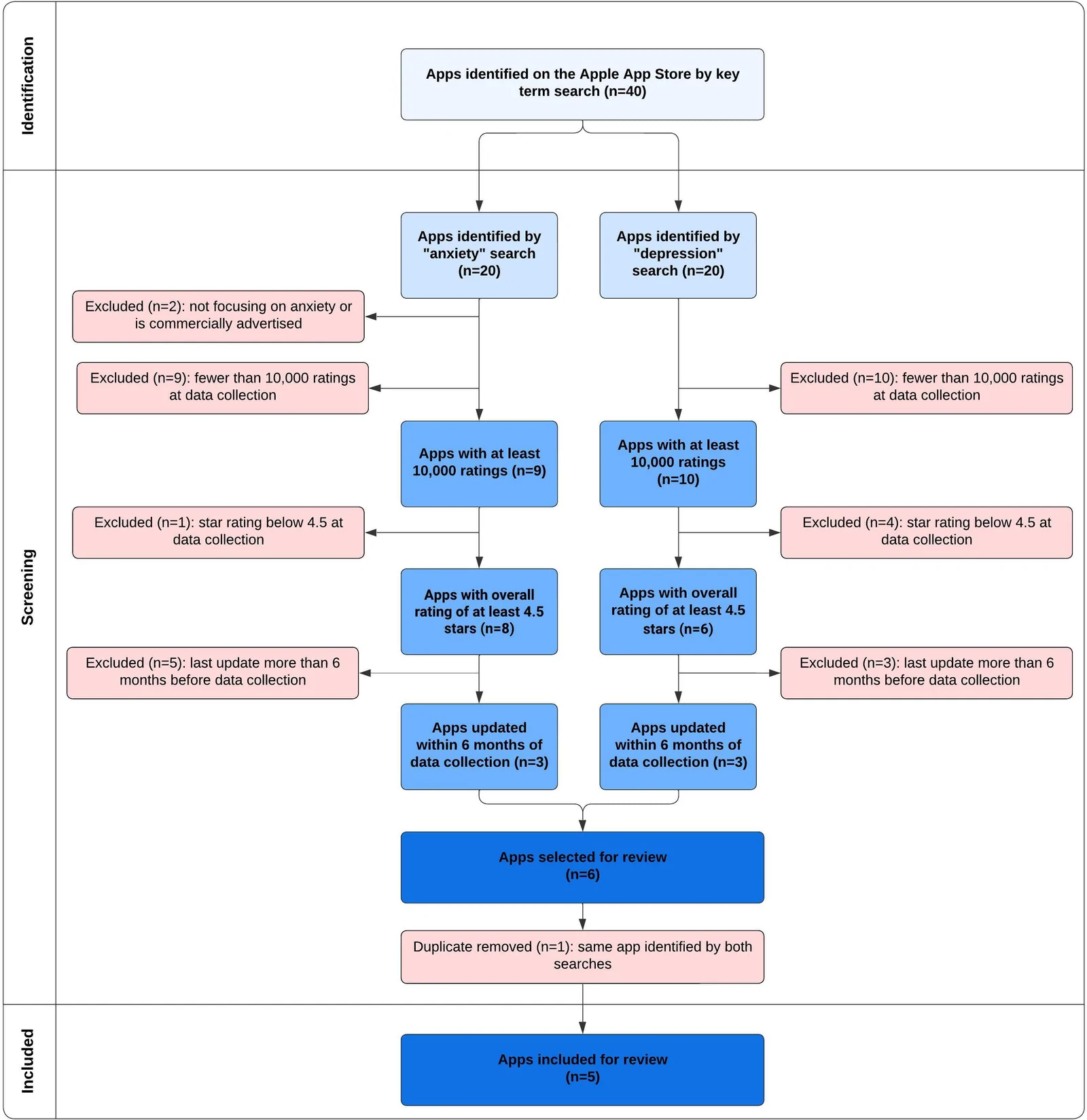
MHapp selection process from the Apple App Store: identification, screening, and inclusion. MHapp: mental health app.

**Figure 2.**
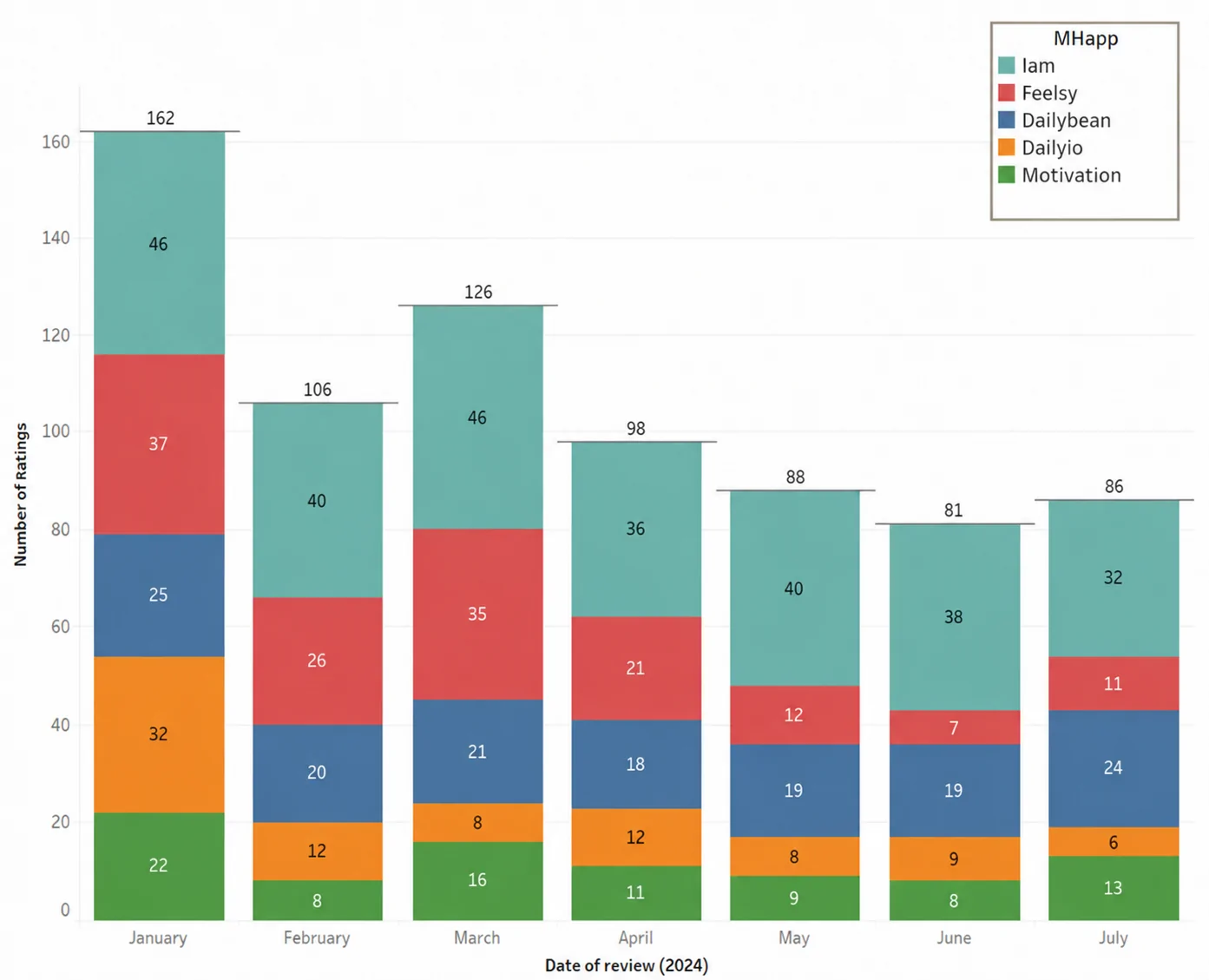
Number of star ratings by app and time. MHapp: mental health app.

**Figure 3.**
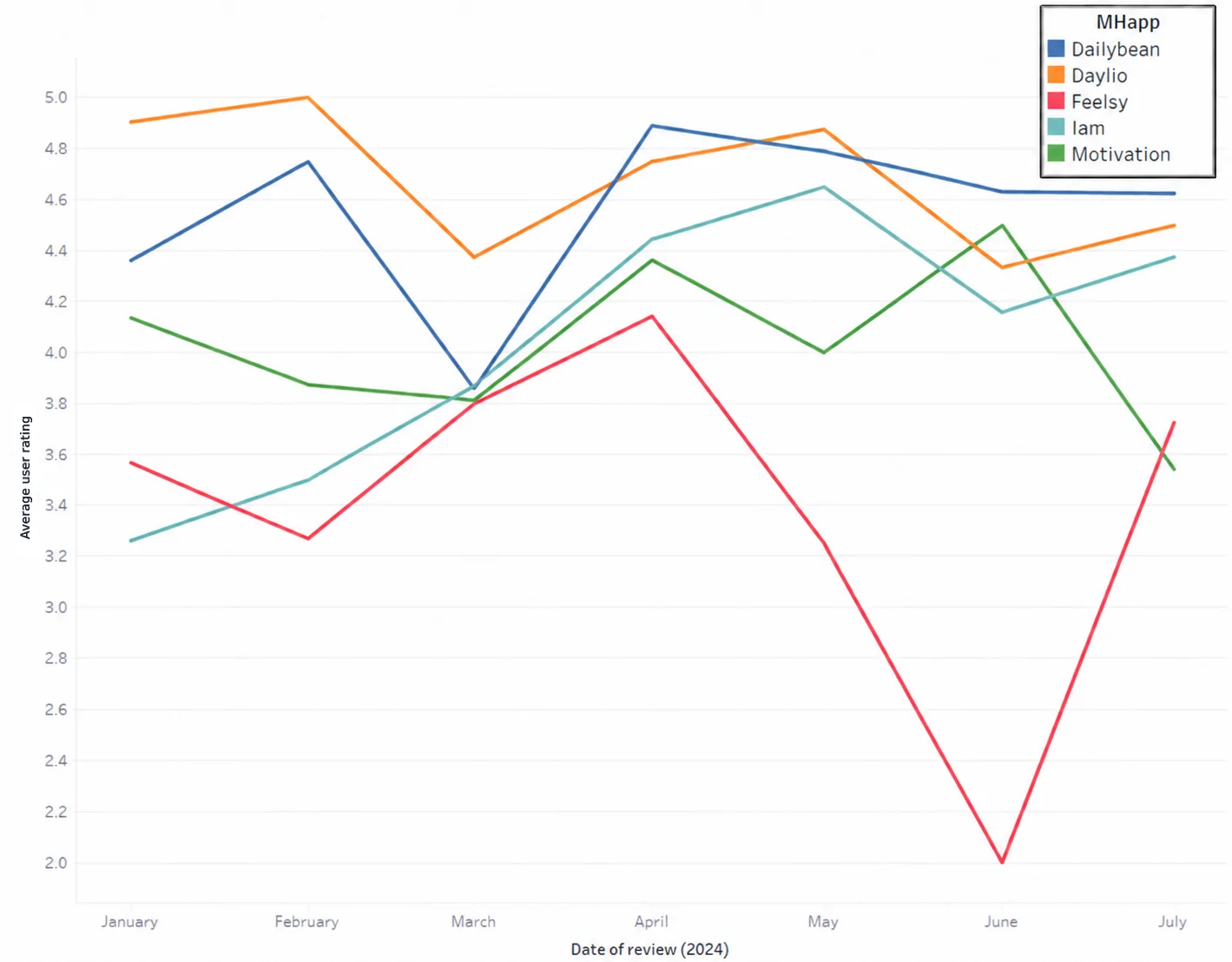
Average star ratings by MHapp (mental health app) over time.

### Thematic Analysis

Feedback varied widely; many users expressed gratitude for the convenience and accessibility of mental health resources provided by the apps. While high ratings and positive comments highlighted the apps’ effectiveness, some reviews—despite their high scores—voiced significant concerns regarding the user interface, pricing structures, features, and overall functionality. This feedback is invaluable for refining the user experience and enhancing the impact of mental health support through technology.

### Theme: Perceived Helpfulness of the Apps

The first theme that was identified showed that positive experiences typically aligned with high ratings. Users described the apps as “helpful,” “fun,” and “recommend,” and emphasized using the apps for emotional support, accessibility to mental health resources, and convenience to their lifestyle. Longer and detailed reviews provided descriptions of the reasons for recommendations and the features that positively influenced the user. Such reviews showcased the reviewer’s ability to reflect and articulate their experiences in a public forum and openly narrate their personal experience.

Using the custom features to add moods, things I’m tracking and more makes it easier for me to understand what was impacting me that day and why I felt the way that I did...

Users with these types of reviews showed special intention and insight into human behavior and psyche. These individuals showcase the unseen capacity for technical literacy, the confidence to share their opinions in a public forum, and the confidence to narrate their experiences openly. Although many raters gave 5 stars to the apps in question, the descriptions of their experiences highlight thematic differences within the overarching theme. These detailed accounts revealed how app use can go beyond simple star ratings and shift to nuanced experiences specific to user type.

Started at a very rough place in my life, dealing with highschool finals and trying to figure out what to o with my life next. i was denied from what i thought was my dream school that i was going to play a sport at, and i spiraled for weeks. i got back on my own two feet with the help of the goal setting this app has, as well as many other features.

#### Subtheme: Personalized App Features Facilitating Self-Reflection and Growth

Many users leaned on specific app features that best catered to support their mental well-being, such as goal-setting help, custom mood tracking, and journaling with minimal effort. Users highlighted its impact on their life by describing how such practices supported them in reflecting on and identifying the root causes of their distress in life. Users noted a growth in mood stability and confidence and added how personalization options for tracking moods, activities, and adding personal notes or photos enhanced their understanding of daily influences on their mental health.

This app has honestly helped change me from behind the scenes, I’ve tried notebooks and rage rooms but this app really helped me reflect on my problems, what causes them, and how i handled them in the past. I’ve been able to learn from and look back on my past self and its amazing to see how much I’ve grown in just 3 months of using the app.

An advertisement-free experience contributes to a more personal and uninterrupted user experience, making users feel valued rather than exploited for profit. Additionally, these apps facilitate personal growth and reflection, allowing users to learn from past experiences and track their progress over time. These aspects underscore the effectiveness of MHapps in providing emotional support and fostering personal development.

This app is amazing and the fact that there is no advertisements makes it feel so much more personal. i feel like the developers have seriously done something so amazing for thousands of people and not just using struggling individuals for a profit is something that no other app ive used has done. thank you!

#### Subtheme: App Features Effectively Used to Manage Clinical Symptomology

Several users explicitly referenced diagnosed mental health conditions such as anxiety, depression, attention deficit/hyperactivity disorder (ADHD), posttraumatic stress disorder (PTSD), or postpartum depression and described using features within the apps that helped them manage their symptomology. Users reported selecting the app after trialing several others before landing on this one. Based on specific features aligning with their symptom needs, users described their perceived effectiveness and how closely feature-symptoms fit is more important than overall app popularity. For example, 1 user listed the helpfulness of writing their feelings and tracking their mood. Goal-setting features were found to be particularly beneficial across several users to manage clinical symptoms as they were used as gentle nudges and structure without feeling burdensome.

I recently got diagnosed with anxiety after struggling with it for many years and downloaded this app after a friend recommended it. Its my favorite app in the world. I frequently need to vent, so I do my brain dumps here, along with tracking my moods and habits. Its simple, only takes a few minutes a day and has helped me discover unknown triggers, as well as the things that help me calm down. I also love the goal feature because it encourages me to complete tasks that I simply may forget or that I normally dont put the effort into doing. 5 stars isn’t enough praise for this app.

#### Subtheme: Personalized Usability and Cognitive Load

Several high ratings were associated with the use of minimal effort using icons and pictures to track their moods. The brief engagement, however, showed a positive effect on completing the daily tasks and goals set by the apps, leading to sufficient symptom monitoring and thus promoting emotional awareness. One commonly praised feature was the ability to customize entries, activities, and add personal notes or photos. This customization helps users understand the daily influences on their mental health and provides a personalized journaling experience. The aesthetic customization options, such as widgets and themes, are appreciated for making an app visually appealing and integrating it smoothly into users’ daily routines. Another appreciated feature was the option to dictate journal entries, which simplified the process and made it more accessible for those who prefer speaking over typing. Users also valued the seamless review of previous entries per month, promoting self-reflection and identifying temporal patterns.

I’ve been journaling for decades, but never as consistently as with this app. I did a lot of customizations that make sense to my lifestyle. But I love that I can record my journal entries verbally from my phone and see the data I track over time. It’s also easy to review previous entries in a seamless way by month. This app is amazing and totally recommend if you want to get into habits, review the day, save for memories, how you have improved in anything, and keeping track of sleep, etc! It doesn’t require a ton of work which is a massive green flag…

Comparing these apps to digital habit trackers, users reported that the absence of streak mechanisms allowed them to track habits and patterns without feeling overwhelmed or inadequate. The app interface was reported to encourage users to engage in a way that feels natural and enjoyable, such as a fun design like a “little bean” each day, which adds a playful and positive element to the experience. This approach helped users look forward to their daily check-ins rather than dreading them. Additionally, the apps provided valuable insights into users’ emotional patterns, helping them realize that their perception of frequent sadness might not align with reality. By offering a more nuanced view of their emotional state, the apps helped users understand that their memories of sad days may overshadow the more neutral or positive days they experience. It emphasizes the importance of self-worth beyond mere streaks, fostering a healthier relationship with goal setting and self-improvement.

Other similar apps like habit trackers start off strong, but I end up feeling really bad about myself when I don’t complete a goal or miss a day and lose my streak. I’m a human. I am worth more than a s***** streak. This app allows me to track my habits and identify patterns without that forced feeling of hustle culture...

Overall, the reviews for these apps supported their user-friendly and empathetic design that helped mental well-being by reducing the pressure of perfection and promoting a balanced perspective on one’s emotional health.

#### Subtheme: Length of Engagement

Users reported both short- and long-term use with the apps that influence their behavior and features perceived effectiveness on their life. Cumulative benefits such as emotional awareness, habit tracking, new habit formation, and coping skills were described by long-term engagers. For example, an app user of 4 years stated how consistent “venting” allowed reflection and emotional awareness, helping them manage their mood and symptoms of depression and anxiety.

Had this app for almost 4 years now, I suffered from MAJOR depression, anxiety and self-esteem issues. This helped me so much, I vented so much and was able to see what my headspace was in my bad times to see what I could do. Super easy, it helped me install healthy habits and better ways to express myself in a non-destructive manner.

Short-term users reported more superficial use, reflecting meaningful everyday use that motivated them to return to the app’s customizable features.

I have been using this app for about 3 days now and it’s improved my writing skill and made me really think about my day and what happened my favorite part is that you can add stuff on and that you can make the beans into cats, planets or whatever it’s super cool I definitely recommend getting the app if your committed to doing this everyday. You can write for the past days that you didn’t do and it takes less then 15‐20 minutes to do!

### Theme: App Frustrations

#### Subtheme: Technical Accessibility

Sustained use of the apps exposed issues related to features and technical vulnerabilities. Some long-term users expressed frustration with changes in legacy features such as changes in notification settings, losing positive motivation through the app, etc, despite troubleshooting attempts.

I love this app and had it for two years. but did change something that they remove notification with affirmation? I use to receive notifications on my phone of these positive affirmations but it has stopped for the past several months and it stop showing up on my screen when lock my phone. I tried everything to receive those notifications again including updating the app, updating phone, turn on all notifications, insolence the phone, restart my phone. I would only receive the notifications if I open the app. But receive none the next day.

Reliability of technical factors was central to users’ continued engagement with the apps. However, updates either altered or removed available options, frustrating users, especially long-term users, who had absorbed the app into their daily routines.

I’m paying for this and can’t get it to operate to my own personalized preferences like I used to. I am thinking of just canceling my subscription. I gave it a good run for years maybe its just run its course and owner is not thinking there are problems bc reviews are good. I had this for years and I think its a great app but I obviously I have a lot of user experience with it to know the tech is remedial and the price increase from 2.99 to 3.99 a month is not worth it if I cannot get it to work like it once did. I hope the update solves my issues but if not, it was good while I lasted!

Users with many years of experience reported frustration and felt the app overlooked core features with its growing popularity. This disconnect led to some users considering canceling subscriptions or deleting the app.

It doesn’t really make any sense that a game that is designed for helping people with frustration is extremely frustrating...

Long-term users showed signs of frustration that they can no longer operate the app according to their personal preferences, which they previously enjoyed. They believe the app’s owner might not be addressing these problems due to generally good reviews, which adds to their frustration. As long-term users with a deep understanding of the app’s functionality, they feel their feedback should be taken seriously.

I liked this app and being able to click and read something affirming. However this 20 a day counter isn’t working, I open the app dozens of times and it’s not counting. Now the newest version prompts me to commit to 3 days WTH!?!...

Users giving 5-star ratings affirmed that the rating was given so their reviews would be read by other users. Some users further expressed their frustrations in their reviews by highlighting loss of control, feature instability, and unresolved bugs, significantly undermining the initially perceived effectiveness and trust with the apps. Interestingly, most developer responses were affiliated with bad reviews with 1-star ratings rather than those with 5-star ratings.

#### Subtheme: Affordability

Affordability was a key barrier to accessing apps over time. Many users appreciated the apps’ features but noted the subscription and paywall to be a huge barrier. Users reported that apps, once successful with their free version, transitioned to create a premium version advertised as having “more features” for mental health. It was noted from the reviews that such advertisements or general liking of the app lure users to buy premium versions.

I dont appreciate beating me over the head with buying premium from the second I download the app. In particular, the 24 hour ticking countdown feels egregious and insensitive. I also noticed you showed me three or four different prices for premium within the first minute of opening the app.

Despite the value in supporting their mental health using such apps, users described premium pricing as excessive. In addition, users emphasized the aggressive upgrading prompts on an app to be demotivating and insensitive, especially toward a vulnerable population.

You are just employing sleazy monetization tricks, and I expected better from a mental health app. And this is coming from a professional app developer who understands your desire to monetize...

Some users reported limited enhancement in their user experience even with the premium features. Unmet needs, cost, and loss of trust led to strong dissatisfaction among several users. In addition, technical failures even after upgrading the app further fueled disappointment among users. Others noted that once the free trial ended, the app features were severely limited, prompting the user to delete the app despite initial positive experience. While some users were willing to pay for their mental wellness, often technical stability and cost value influenced user retention with subscribed apps.

I really thought that at least some of the features would be usable after my free trial but I was wrong. I could save anything after my free trial and the app was just pointless so I deleted it.

#### Subtheme: In-App Advertisement

In-app advertisements were reportedly disruptive, breaking the emotional flow and mental wellness experience on the app. Users stated the advertisements undermine the supportive environment and can be problematic during stress or heightened anxiety. The presence of advertisements can also lead to a cluttered interface, making it harder to navigate and use an app effectively. This can be especially frustrating for users who rely on an app for daily journaling, mood tracking, or goal setting.

For the free version, requiring daily, very long ads to track my day is not great for my mental health and causes great frustration. The ads alone are why I skip many days.

Moreover, advertisements can make an app feel less personal and more commercial. Users feel that their struggles are being exploited for profit, which can diminish their trust in an app.

Recently in the past few months I’ve had to watch an ad for every day i wanna log something and its frustrating. If i was able to pay 5 dollars to get rid of ads than I would, but that’s not an option. I HAVE to pay for premium, and I am not willing to spend 30 bucks a year to get rid of ad and gain a bunch of features I m not going to use.

Many users appreciated apps that offer an advertisement-free experience, even if it means paying for a premium version. The absence of advertisements allows for a more immersive and supportive environment, helping users to focus on their mental wellness without distractions. Overall, user reviews that discussed in-app advertisements indicate that the volume and placement of such advertisements are crucial in shaping user retention and overall engagement.

### Theme: External Motivators

Several user reviews highlighted members of their community that played a role in motivating them to adopt and use these tools. Apps were reportedly recommended to users by friends, family members, and therapists or care providers. Such endorsements were reflected in the reviews and contributed to faster adoption. Users showcased eagerness when health care providers recommended MHapps and often reflected on certain features that they used that supported their provider meetings.

My therapist told me about this app, and I haven’t looked back. It really does help me. Especially when I am having one of those extremely dark moments.

Additionally, encouragement from family and friends can play a crucial role in adoption and consistent use of mental wellness apps. When family members recommend an app, it often holds a sense of trust and reliability, making individuals more likely to give it a try. Knowing that someone has benefited from the app can provide an incentive to explore its features and integrate it into their daily routine.

This app truly changed my life before this app I was very sad until i was recommended this app my aunt she used this app and said it helped her mental health. I didn’t believe her until I saw it for myself this app is truly amazing words cannot describe how this app is very helpful

### Theme: Feature Requests

User reviews frequently included general comments on the app use followed by feature requests showcasing a desire for continued use through app improvement. These requests provided insight into how current users are incorporating features into their daily lives and tap on user preferences for app improvement. For example, users expressed a need for more dynamic content to keep them engaged, such as customizable options based on their daily mood or experiences indicating evolving user needs.

One feature request - please add support for landscape mode orientation on iPhones. Sometimes I connect my phone to an external display and keyboard specifically to type up entries.

Personalized features were a recurring request in user feedback directed to developers. Users appreciated the ability to tailor the app to their specific needs, whether it is through personalized stickers, higher limits on photo uploads, or support for different device orientations. Additionally, requests for features like quests or daily challenges suggest that users are looking for interactive elements that can motivate them without additional costs.

I would also like to see like a thing to customize when u say how ur day is if (for example) u say your day was great then u customize specific thing that show up like how u feel but it only gives u happy options bc u said u had a good day.

Requests for daily questions, higher limits of media uploads, and optional quests or challenges suggested users were seeking interactive elements that can enhance their engagement. Moreover, the significance of these feature requests lies in their potential to improve user satisfaction and retention.

Please consider adding a higher limit for the amount of pictures you can put in a day because sometimes I have days with like 4 or 5 pictures that I really want to remember but the app only lets you put 3 pictures.

Frequently, developers responded publicly to the feature request by inviting the user to share their feedback via email, signaling a supportive approach and openness to cocreation of the app. Such practices not only improve the overall effectiveness of the app but also foster a loyal user-base that feels heard and valued.

## Discussion

### Principal Findings

This study examined user-generated reviews to reflect on user insights that highlight facilitators and barriers to user experience with highly rated MHapps. The study highlights how factors such as usability and quality assessment of app engagement go beyond the static star rating scale of app-store MHapps for its consumers.

By means of a user-centered qualitative analysis of reviews, this study situated individual reflections within broader contexts of a user and their described environment. User reviews provide a passive window into the privacy concerns about individual data use, demonstrating that user experiences with MHapps are driven by complex interactions with app design, social support, trust, personalization, technical ability, and affordability. Collectively, the most prominent insights were with individual app experiences extending into collective peer-informed communities and users acknowledging intentional app design for long-term engagement. Tensions related to cost-affordability and commercialization represented the complexity of users’ app experiences and showcased meaningful facilitators and barriers to its use. The study showcases how users can transform individual opinions to drive app engagement and growth in design elements and that an app’s perceived effectiveness hinges on the integration of individual, interpersonal, and social elements, as well as functional and design dimensions of technology. Altogether, the findings compel a reevaluation of how MHapps usage is assessed and emphasize the importance of lived experiences as a lens for understanding digital health tools.

One of the appreciated findings was the degree to which users transform individual app experiences into collective experiences. This finding responds to research questions (1a) How does a virtual user interact with apps designed for depressive or anxiety-like symptoms? and (1b) What factors encourage or discourage users’ motivation to engage with the apps? The user reviews represented a community with high motivation to improve or maintain their mental well-being and support others who may have similar experiences. Through a narration of their own mental health journey and describing the positive impact from app recommendations, it showed that app interaction often goes beyond an individual transactional exchange with the app, extending into a distinct social and relational form of engagement. Such open discussion of personal wins and struggles created an informal peer network around the app itself, thus extending it beyond an app’s initial design vision. This showcased that there is more to the informal peer community ecosystem that drives users indirectly to app use by factors such as perceived anonymity and shared experiences. These findings extend the understanding of app-review context specifically and have important implications for how MHapps are conceptualized in research and practice. Furthermore, it highlights the importance of intention and personalization in MHapps, demonstrating that users seek tools that cater specifically to their needs. These features not only improve the user experience but also support mental health by providing a sense of control and personalization. Users’ willingness to be vulnerable in their reviews highlighted their desire to help other users who may be struggling with similar conditions. These findings are aligned with research on peer support and online forums, where anonymity and relatability have shown improved help-seeking behaviors [53]. This study’s findings support the existing literature and extend it, highlighting an unintended form of social interaction within informal communities, which suggests that it is an underexplored resource to understand user engagement.

Responding specifically to research question (1b), the motivational role of social networks, such as family recommendations and peer encouragement, was a notable dimension of encouragement of app use. Such motivation influences user interaction with the apps beyond the features occurring during initial use and its continued use. Socially withdrawn and low-motivated individuals responding to an external prompt in initial app use is clinically relevant, especially for people experiencing anxiety and depressive symptoms. The results showcase how user experience with MHapps is not only characterized by its star rating and features but goes beyond—driven by users’ expectations, trust in technology, and emotional vulnerability. Users consistently reported and acknowledged recommendations from trusted sources like family, friends, and health care providers. Some even showed persistence through technical issues since the app recommendation came from a trusted source, indicating that indeed social context influences user expectations and trust in technology. This has meaningful implications for how developers, clinicians, and researchers can conceptualize app research that goes beyond in-app use and consider user commentary and shared experiences as valuable sources of data. Implementation science frameworks emphasize the importance of stakeholder involvement, and this study shines light on the advantages of MHapp engagement when trusted sources, especially health care providers or clinicians, recommend certain digital tools [54]. Furthermore, users frequently used reviews as a medium to request new features, highlighting their desire for continuous functional refinement and personalization beyond the MHapp’s current offering. This theme adds its unique perspective to research question (1b), suggesting user motivation stemming from app evolution to remain engaged. This finding reflected users’ dynamic patterns of engagement, suggesting that users valued continued app evolution as they simultaneously appreciated existing features that furthered their mental wellness. These requests, ranging from adding personalization to tracking options, offered a direct user account of unmet expectations or gaps and provided valuable insights into user needs and preferences. These requests could be collectively used to guide developers for targeted refinement rather than adding general complexity, enhancing overall user experience and satisfaction. This is consistent with literature on user-centered design that emphasizes progressive and feedback-based app development as a critical focal point to app sustenance [55-57].

Continuous engagement with an app can lead to self-reported mental health improvements, while maintaining app functionality and support is essential for user satisfaction. However, in-app advertising practices and subscription prompts complicated experiences, especially for long-term users of the app. Users often find that advertisements break their concentration and interrupt their flow, making it harder to focus on their mental health activities. This reinforced concerns with the ethics overlaying the commercialization of digital technologies, especially when monetizing tactics conflict with user expectations. MHapps are designed and marketed to provide a calm and supportive environment, but aggressive premium or subscription prompts can disrupt users’ motivation and be counterproductive in moments of stress. The abrupt loss of functionality due to affordability limitations or the end of a free trial created a loss of engagement, with users describing the app as “pointless” once core features were inaccessible. Dissatisfaction that persisted beyond initial subscription prompts was relatively due to premium features underwhelming in relation to their cost and associated technical failures. Users revealed that transparency in added benefits with paid subscriptions can reduce the negative impact of the paywall. These findings were important to note for research question (1b) as affordability can play an added role in user encouragement to engage. There is a rising number of MHapps and with that comes a heightened concern about data and security risks [58]. Trust and affordability are pivotal and complex components of user experience that require independent analysis directly with active users. Resonating with broader digital health literacy research, the implications of these findings are 2-fold: first, a need for interpersonal interventions focused on making MHapps easily accessible and affordable; and second, increasing awareness of data use, making systemic-level policies, such as the terms of use, privacy policies, and data security architecture, more transparent and easily accessible by app users. Interestingly, app usability can elicit paradoxical responses due to intersections within themes and subthemes, such as the desire for a simple interface versus the need for advanced features, or personalization versus privacy concerns. It also extends the critiques of the “attention economy” in the digital and information age and ethical frameworks that govern health app design and distribution [59].

Several reviews uncover that MHapps do not need to be overly complex to be effective. This finding directly responded to research question (2) How does a virtual user assess the effectiveness of an app that is advertised to manage depressive or anxiety-like symptoms? where users assessed effectiveness through the simple, yet thoughtfully designed features that can provide substantial support and encourage users to continue using the app consistently. The apps emphasized repetitive interactions with streaks and tracking, demonstrating tangible changes in emotional regulation and self-reported mental health conditions such as depression, anxiety, ADHD, insomnia, etc. The duration of app usage, whether long-term or short-term, played a crucial role in the benefits of users’ experience. The ability to set and track goals, monitor moods, and reflect on progress can make a significant difference in a user’s mental health journey. This simplicity, combined with effectiveness, ensured that users felt empowered and supported, making it more likely for them to stick with an app over time. The simplified function with icons reduced the burden for many users to spend extended periods of time with an app. Yet, the reduced engagement time encouraged continued use. These findings consistently answered research questions (1b) and extend into answering research question (2) by demonstrating that users evaluate effectiveness through features but also through design that integrates functional, social, and emotional dimensions simultaneously. Simplifying the user interface positively affected behavior, especially for users experiencing anxiety, demotivation, and disinterest. Design elements such as brighter colors, visual aids, and language motivate users to engage and benefit from its features [60]. Understanding these aspects can lead to an improved app design that better meets user needs and preferences. However, despite high star ratings, user experiences with MHapps varied significantly due to factors such as user interface design, personalization options, in-app frustrations, and technical issues. Users with higher technical understanding and confidence tend to navigate apps more effectively, providing detailed and positive reviews, while negative feedback often reflects unmet expectations, costs, or cultural misalignment with app offerings. Variation in experiences accentuates the importance of user-centered design and continuous improvement in meeting diverse user needs [61]. Users who encountered misaligned content or design features were effectively demotivated from using the app and critiqued its effectiveness on their mental wellness. Such users made their difficult position amply clear in their reviews. Ultimately, the findings underscore the value of designing effective MHapps that are both user-friendly and impactful.

This study contributes to the growing body of literature by exploring digital interventions for mental wellness using user experience as a central determinant of app engagement and perceived value. It establishes a foundation to understand user-centric functionality, engagement, and success through user perception. With its noted advantages, including a comprehensive approach and exploration experience, the results have several promising implications for future researchers. By assessing the App Store reviews in-depth, the findings highlight how personalization, usability, interface design elements, and affordability can shape users’ attachments with apps and embody them in their daily routine. The results demonstrate that although understanding of user experience with MHapps may be a complex mechanism, the app does not need to be complex but focus on reliability, adaptation, and sensitivity to the daily needs of an individual needing emotional support. Users often are driven to affordable, low-effort features and simple designs that reduce cognitive and emotional burden. Overall, these findings showcase the importance of user-centered and theoretical grounding methodology to assess app selection and design features beyond star ratings. This study centers on the lived experiences of users and offers actionable insight for future studies as well as for developers and clinicians seeking to identify technical design elements that meaningfully support mental health. With more evidence to bolster current efforts, this study will attract future researchers and open more branches for theory derivation to better understand the abstract nature of the interaction between digital media and human behavior.

### Study Limitations

While qualitative analysis of user reviews can provide rich, nuanced insights into user experiences and preferences, this study has limitations when interpreting the findings. The findings reflect the researcher’s interdisciplinary background and analytical lens that shaped how codes were clustered into themes. The coding variability was addressed through systematic documentation of the coding process and consensus development between researchers. However, for this study, theme construction prioritized depth over breadth, and thus its interpretive approach does not address broader engagement trends. Future studies may cultivate it by adding quantifying methods such as in-app analytics and retention data.

This study limits its analysis to 5 apps, which constricts the generalizability of the findings. The analysis was limited to the Apple App Store ecosystem, which constricts the transferability of findings on Android users. While the utility of Apple phone users in the US context is higher than Android, the current findings may not fully represent the wider user community [62]. These reviews were analyzed without access to demographic or clinical verification, limiting the ability to contextualize experiences by age, sex, gender, or clinical severity. Since these reviews consisted of self-reported app use, the self-reported diagnosis was used as adjuncts. Lastly, the research is subject to selection bias as only users who actively leave written comments represent the subgroups, in which the silent users are unaccounted for, thus leaving a broader user base. Nevertheless, this study provides a foundation for scaling analysis to larger and more diverse databases.

Despite these limitations, the findings provide insight into such unguided apps’ access, use, and overall experience, thus reflecting real-world cases on unguided apps as opposed to formal, clinically driven, stand-alone apps. Qualitative analysis remains a valuable tool for understanding user perspectives and improving app design. Balancing its strengths with these limitations is crucial for practical application in research.

### Conclusions

The study findings challenge the stand-alone MHapp interventions and showcase that apps operate within a broader ecosystem of social interaction, trust, design interactions, technology use, digital literacy, and affordability. It also emphasizes shifting the lens of MHapp evaluation from a simplified question such as “is an app effective?” to understand context and use mechanisms that can support individual mental health. Such user-centered research can allow apps to be a complementary tool rather than a mere replacement of clinical support. The study also recognizes that users need to control their experience through personalization but also transparency of data use. As the use of MHapps grows, it is important to consider data security and privacy as critical determinants of user trust and retention. This study also illuminates the heterogeneity of user experience hidden behind high ratings and reveals critical gaps in affordability and the trade-offs of commercialization through its aggressive monetization practices. These seekers of health apps need accessible mental health support; thus, while businesses need sustenance, they must align with the context of their health needs. This study calls for further work in examining the ethical implications of in-app advertisements, especially when they can be counterproductive for MHapp users. Additionally, future studies may assess how MHapps can ethically develop pricing models that limit their emotional impact on users that benefit from such tools.

MHapps represent an unprecedented number of available digital tools. However, to realize their true potential requires researchers to move beyond technical efficacy and understand the social and functional dependent systems surrounding users. This study provides evidence that users are evolving and value simplicity in functions, respect for their data, accessibility, and affordability. It demands a second glance by researchers, clinicians, and developers to recognize the information supplied by the community of users under MHapp user reviews and create digital interventions that meaningfully support mental health care.

## Supplementary material

10.2196/97517 Multimedia Appendix 1 Coding structure of themes, subthemes, and example codes.

## Abbreviations

ADHD: attention deficit/hyperactivity disorder
CBT: cognitive behavioral therapy
CFAA: Computer Fraud and Abuse Act
IRB: institutional review board
MHapp: mental health app
mHealth: mobile health
PTSD: posttraumatic stress disorder
SOP HRP: Standard Operating Procedures Human Research Protection
