# Supplementary material for: Using App Store Reviews to Explore User Experiences and Perceptions of Mental Health Mobile Apps: Qualitative Study
Source: JMIR Form Res. 2026 Oct 8;10:e97517. doi: 10.2196/97517 (PMC13648948; doi:10.2196/97517)
Supplement: Supplementary file 1 [file formative-v10-e97517-s001.docx]

**Supplementary Material 1. Coding Structure of Themes, subthemes and example codes.**

| **Theme** | **Sub-Themes** | **Example Codes** |
| --- | --- | --- |
| Theme 1: Perceived Helpfulness of the Apps | Personalized app features facilitating self-reflection and growth | understanding patterns in mood, discover triggers, tracks self sabotaging behavior, maladaptive habits, changes perspective on life, improves attitude, better person, happier, accountability, productive, habit builder, installed healthy habits, helps reflect and calm down, helps regulate emotions, helps with emotion tracking, goals, help feel better, growth noticed, improving life, better aid in sleep tracking, healthier sense of self |
|  | App features effectively used to manage clinical symptomology | positive impact on mental health, manage mental health, helps with anxiety, depression, ADHD, stress, anger management, aggression, mood disorder, helps with social engagement issues, self-esteem issues, good for emotion tracking, great for mood trends, comforting over other habit apps, helps with fear, helps be on time, reports useful for therapist, therapist use, shown to doctors, uses with therapist, more useful than therapist, need for mood tracking ,tracks sickness periods, diet and mood, chronic pain, stress relief , great for anxiety |
|  | Personalized usability and cognitive load | easy UX, simple, simple and effective UI, extremely approachable, aesthetic UI, cute UI, adorable UI, fun UI and UX, engaging UI, interactive UI, beautifully designed app, visually pleasing formats, customization, customizable, custom designs, custom icons, different fonts and colors, themes, personalization offered, customizes to user needs, flexible, icons and emojis, emoji use, pictures for tracking, picture use, adds photos, notes and pictures, allows photos with entry, customizable entry, one click feeling tracking, quick, three minutes out of the day, fast logging, accessible, reminders, reminding to check in, gentle reminders, notifications not annoying, never feels forced with reminders, fits lifestyle, easy even as non-premium member, good for beginners in self-care |
|  | Length of engagement | daily usage, daily tracking, daily record maintained, daily app use, consistent use for journaling, continued use of app, long-term use, long-term user, over a year use, used over a year, entries for over a year, used since 2017, since 2018, since 2019, since 2020, used for years, few years used, 2 year use, 3 years, 4 years, 5 year use, 6 years use, used everyday for four years, consistent use for 4 years, 1900 day streak journaling, 960+ day streak, streaks helpful |
| Theme 2: App Frustrations | Technical accessibility | settings confusing, interface confusing, too many steps, app getting complicated, not accessible, serious update needed, boring, visuals not unique, icons UI not good, limited tracking features, word count limitations, cannot customize name/background, unable to change activity name, cannot add own quotes, no feedback for mood entries, surface level emotions only, bugs, widgets not working, glitches, app resets during update, notifications not working, not receiving notifications, cannot log in, past data vanished, data loss, lost journal entries, unsaved data lost, cannot save after free trial, iCloud backup needed, limited free version functionality, constant app updates, confused with interface , unavailable features, limits retrospect on journal entries , less return with more engagement ,pop-up during use, annoyed, expectation to write own quotes that is under premium ,fantastic until paid for it , widget not working, no technical support ,technical difficulties ,technical difficulties ,resolved via developers , frustrated with the notifications ,premium needed for any features ,updates broke widgets ,app now useless ,app now useless ,technical issue unresolved ,unresponsive support team |
|  | Affordability | unable to afford premium, cannot afford, steep subscription price, too pricey, very expensive, expensive themes, greedy subscriptions, too many premium features, most features paid, everything under subscription, features locked behind premium, drained by features locked, premium inaccessible, dislike paid features, paid features not useful, subscription model disliked, not one-time payment, automatic billing concerns, fear of sudden billing, charged after cancellation, trial limitations, need VIP beyond trial, pressure to pay |
|  | In-app advertisement | frustrated with ads, ads are annoying, too many ads, ad bombardment, ads after every action, longer ads duration, ads increase stress, not relaxing due to ads, ads tied to functionality, watching ads to earn currency, engagement tied to ads, dislike ad-based currency, ads interrupt experience, ads reduce enjoyment, ads defeat purpose, misleading advertising, less exciting due to ads, paid features with increased use, dreads ads, now ad duration is longer , ads annoying , ad amount is high , after 1-2 months ads showed , does not like ads, advertisement misleading ,dislike ad watching to earn ad currency , recent update includes ads, app use associated with ads, contradicts to its purpose , backfiring, ads to pay ,ads in use , ads affects mood ,free app with ads, stuck on ad, unreasonable ,ads, 40 sec ads, cannot navigate grief ,cannot navigate grief ,pop up ads, cant read without an ad, ads every 5 seconds, quality lowed with ads ,ads between quotes ,pops every 2 mins,15 sec ad, loud ads |
| Theme 3: External Motivators |  | recommends to friends, recommend to family, high recommendation, therapist recommended, therapist suggested, friend recommended, peer sharing, uses with friend, reports to therapist, shown to doctors, social sharing, community expression, friend recommendation, shares with friends and family , has shown data from it to doctors and peers, family use ,someone recommended, gets compliments recommends to family ,sent to friend, family supported ,grandmother and mother ,recommended by friend ,recommended by counselor, recommends to patients, therapist recomended,recommends to other |
| Theme 4: Feature Requests |  | theme request, request feature discussion, remove ads, reduce ads, cheaper premium, one-time payment option, more free features, fewer paywalls, better customization, infinite scrolling, Apple Health integration, improved data backup, iCloud backup, add own quotes, better mood feedback, deeper journaling prompts, improved UI/icons, fix bugs, simplify interface, remove religious content, hide religious affirmations, improve accessibility, more engaging updates, remove app currency system, more photo options, better analytics, trending reports, longer free trial, clearer trial transparency, request lower prices, request categories, add dark mode, new feature requested, religious activities section recommended, complaint on no feedback for sad mood , new feature request , wants connection to Apple Health to track sleep |
